# Optimized decomposition of fresh tomato remnants in facility soil

**DOI:** 10.1016/j.heliyon.2024.e29590

**Published:** 2024-04-14

**Authors:** Jingmin Zhang, Wenxia Yang, Di Feng, Xiaoan Sun

**Affiliations:** Weifang University of Science and Technology/Shandong Facility Horticulture Bioengineering Research Center, Weifang, Shandong, China

**Keywords:** Facility soil, Tomato remnants, Decomposition rate, Organic carbon breakdown rate

## Abstract

To return vegetable remnants to soil in situ and understand parameters that determine their decomposition efficiency, the tomato remnant length, soil moisture, soil temperature and dosage of a microbial decomposer (MD) have been evaluated through a laboratory experiment using a nylon mesh bag in this study. The results showed that the residual remnant weight, and total carbon content increased 28.49 % and 32.65 %, respectively with two different remnant lengths (∼0.5 cm and ∼2.5 cm), while the decay rate and organic carbon breakdown rate decreased by 6.14 % and 7.48 %, respectively. When the relative water content in soil increased, the residual remnant weight and total carbon content first decreased and then increased, while the trend of the decay rate (16.94 % with 80 % soil water content) and organic carbon breakdown rate (9.96 % with 60 % soil water content) were opposite. At a high MD dosage (7 % or 9 % of the total compost weight), both rates of remnants were greater than those at the low dosage (1 %), with an increase of 38.63 % or 36.19 % and 15.89 % or 15.78 %, respectively. With an increase in soil temperature, both residual remnant weight and total carbon content decreased first and then increased, while both decomposition rate and organic carbon breakdown rate increased first and then decreased by 27.35 % and 22.78 %, respectively at 45 °C, compared with those at 30 °C. It was concluded that the decomposition rate was significantly correlated with the remnant length and the MD dosage, while the organic carbon breakdown rate was significantly associated with all four parameters evaluated. The optimal decomposing efficiency was achieved through cutting tomato remnants to a length of ∼0.5 cm, maintaining soil relative moisture content at 89 %, keeping soil temperature at 50 °C, and adding 7 % microbial decomposer MD to chopped tomato cuttings.

## 1 Introduction

Facility vegetable production has rapidly expanded to more than 4 million hectares in China for the last decade and become an offseason vegetable industry. With a massive production of fresh produces in different enclosed facilities, the treatment of vegetable remnants in a large quantity has also become an issue or a nuisance due to lack of a recycling program in the remote rural areas, therefore, returning vegetable remnants to the facility soil is the only option available for majority of vegetable growers in China. So far, plant remnants can be returned to the field directly or through an off-site process of compost. The later often requires more efforts and investment on transportation, human labor and compost facilities. Due to a large workload and high cost, vegetable remnants are generally directly returned to soil in situ in the current facility cultivation practice.

Soil microbial communities play a key role in the decomposition and the formation of humus compounds [1]. Degradation of plant remnants is a biochemical process involved with various microbes and in their collective interactions, therefore, the selection of multiple microbial decomposers is crucial for degrading plant materials efficiently [2]. Composite bacteria as potential decomposers can be formulated through a combination of single strains or via a direct use of soil microbes isolated from a preliminary screening. It has been a common approach to isolate composite bacterial decomposers from the special environmental materials. Daza et al. isolated and screened strain P1C with enzymatic activity from compost piles to improve the decomposition rate of sugarcane residues by helping to degrade lignocellulose components, starch, and protein [3]. Tsegaye et al. isolated two new bacteria from wood eating termites and it can effectively remove lignin from rice straw [4]. All these findings have suggested that the direct isolation of composite bacterial strains had a better synergistic effect of various and stable microbes on the efficacy of the bacterial decomposition.

The freshness of plant remnants and the plant species affect the decomposing process. Aulakh et al. found that adding wheat residues and green manure to flooded rice is more effective in maintaining high rice yield and increasing carbon and nitrogen content than simply adding wheat residues, due to a more efficient decomposition of plant remnants [5]. Barel et al. found that the lower the C/N ratio is, the higher decomposition rate of plant residues should be obtained [6]. Green manure residues have high carbon and nitrogen and dissolved organic matter (DOM) potential, due to the relatively low carbon and nitrogen content in grain residues, which accelerated mineralization in the decomposition process. However straw residue has the high C/N ratio and mineralization is slow [7]. So green manure is more prone to decay than wheat residue.

Soil chemical, physical and biological properties are important external factors affecting the process of the plant decomposition. A high soil bulk density slows down plant remnant decomposition ^[^ [8]， [9]^]^ and plant remnant decomposition rate was positively correlated to soil available phosphorous, available potassium and soil temperature [10]. The decomposition effectiveness in mineralization depends on soil water content and decomposition phase [11]. When soil moisture levels are within 50–60 % of water filled pore space (WFPS), the decomposition rate is typically the highest [12]. Furthermore, Plant remnants incorporated into soil can absorb water from the soil, which create microenvironments for microbial decomposers, which creates a “sponge effect” that can be beneficial for microbe-driven decomposition [13]. Optimizing environmental conditions has proven to boost the decomposition plant remnants [1].

With the vegetable production in facilities, it is more practical, feasible, convenient and cost effective to return fresh vegetable remnants in situ due to a manageable nature of vegetable production in greenhouses in which the most conducive conditions for a rapid decomposition of tomato remnants are achievable and the in-situ returning more acceptable. Therefore, this study aimed to evaluate the effect of the cutting length of tomato remnants and other decomposition conditions such as soil water content, soil temperature and various dosages of MD on the remnant decomposition rate and provide an optimized decomposition conditions for returning tomato remnants to facility soils in situ.

## 2 Material and methods

**Isolation and preparation of compost microbial decomposers:** 120 soil samples were collected from facilities located in Shouguang, Shandong where tomato remnants had been returned to the soil for more than 2 years. Four sets of the composite microbial decomposer (MD) were selected from those soils that had shown the best efficacy in degrading filter papers through 5 generations of the microbial decomposition [14]. The MD mixture was chosen to be used through this experiment and its main microbial species/strains were analyzed through a genome deep sequencing along with a colony forming unit (CFU) of bacteria in 4.33 × 10^6^ and of fungi in 1.31 × 10^4^. To enhance the degrading efficacy of MD, 50g original soil for MD isolation were added to 1L peptone cellulose substrate (PCS) broth (peptone 5g, enzyme mother extract 1g, NaCl 5g, KH_2_PO_4_ 0.25g, MgSO_4_.7H_2_O 0.01g, CaCO_3_ 3g, dissolved in 1L water) with some 0.5 cm-long vegetable cuttings (1 % of total tomato remnant dry weight). The cultures were incubated for 3 days on the shaker at a speed of 120 rpm. The enriched microbial suspension was the diluted 1000 times for further use.

**Materials:** The bulk density of tested soil was 1.23 g/cm^3^ with a 1.83 % total carbon and 0.23 % total nitrogen. After being dried in the air, soil samples went through a 2 mm-mesh sieve and divided into several portions (500g) in a 20 × 10 × 8 cm black plastic container separately. Fresh tomato remnants containing 85.00 % water and 35.13 % organic content were return to the field at a rate of 120ton per hm^2^, approximately 24.4g per 500g soil with a 3.66g dry weight and 1.29g total carbon content. All sample remnants were chopped into pieces according to the experimental design, weighed (24.4g), and placed in a 60-mesh 20 × 10 cm nylon bag.

**Methods:** The cutting length of remnants, soil relative water content, soil temperature, and the amount of the microbial composers are regarded as four key factors affecting the decomposition process once tomato remnants are returned to the soil. Tomato remnants were cut to a length of 2 cm, soil relative content kept at 100 %, soil temperature maintained at 40 °C, and 2 % of the microbial decomposers added were set up a control (CK) treatment to evaluate the effectiveness of other parameters of interest. Five tomato remnants lengths were cut (∼0.5 cm, ∼1 cm, ∼1.5 cm, ∼2 cm, and ∼2.5 cm). The relative water content of the soil was maintained at 60 %, 70 %, 80 %, 90 %, and 100 %, respectively, while the soil temperature was kept at 30 °C, 35 °C, 40 °C, 45 °C, 50 °C, 55 °C and 60 °C as the treatments. A 1 %, 3 %, 5 %, 7 %, or 9 % freshly cultivated decomposing microbial suspension was added to the chopped tomato remnants, respectively before returning the prepared composts to the facility soil.

To ensure an even distribution of the water moisture and microbial decomposers, half of the soil sample (500g) was placed onto the bottom of the testing box mentioned earlier with fresh tomato cuttings in a nylon net bag that were sprayed on top of the soil. Half of distilled water mixed with the decomposing microbial suspension was then evenly poured on the surface of tomato remnants in the nylon net bag, the remaining 250g soil spread on, and the rest half of distilled water and the decomposing microbial suspension poured into soil. The test box was kept in the incubators set at different temperatures for 15 days, during which the highest decomposition rate of fresh plant remnants was observed from 10 to 15 days. Every treatment was repeated 3 times.

At the end of the incubation, the nylon net bag was taken out, and the soil on the surface of the net bag was carefully washed away with distilled water. The nylon net bag and tomato remnants were dried in the oven to the constant weight and weighed to obtain the weight of the remaining remnants (g). The total carbon amount and its percentage of the remnants were determined by the potassium dichromate bulk weight method and external heating method.

Remnants decomposition rate (%) = (dry weight of remnants before incubation - the residue of remnants after incubation)/dry weight of remnants before incubation × 100 %.

The total carbon amount of the remaining remnants (g) = Total carbon content of remaining remnants × weight of the remaining remnants.

Organic carbon breakdown rate (%) = (total carbon content of remnants before incubation - total carbon content of remaining remnants)/total carbon content of remnants before incubation × 100 %.

**Statistical analyses:** Excel 2010 was used for the data collection and sorting, SPSS 20.0 software used for single factor analysis of variance pertaining to the decomposition rate and organic carbon breakdown rate equation fitting, and for the principal component analysis of the organic carbon decomposition rate.

## 3 Results and discussion

**The microbial decomposer:** One of the most effective MDs was determined through the initial screening and evaluation. The mixture of the MD contains both fungi (36.02 % *Aspergillus*, 32.16 % *Mortierella*, 12.73 % *Myceliophthora*, 7.54 % *Mycosphaerella*, 7.00 % *Acremonium*, and 4.55 % others) and bacteria (10.43 % RB41, 8.82 % *Nitrospira*, 6.03 % *Sphingomonas*, 5.36 % *Bacillus*, 3.44 % *Chryseolinea*, 3.02 % *Pseudomonas*; 2.96 % H16; 2.86 % *Nocardioides*; 2.75 % *Steroidobacter*; 2.73 % *Haliangium*; and 51.58 % others) according the genome deep sequencing data. Main fungal genus was clearly identified while a large portion of bacteria remained unknown due to their extremely low titer and trivial function in the whole bacterial community.

**Fig. 1:**
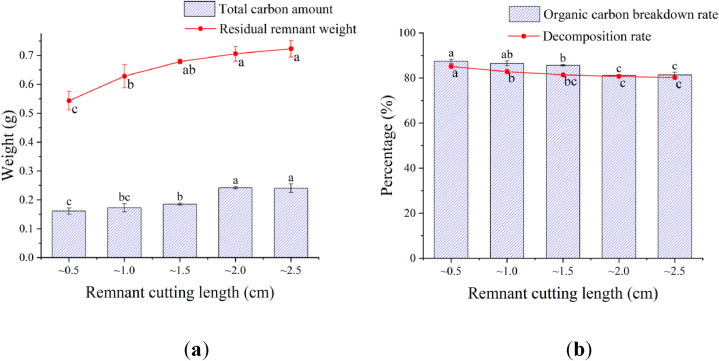
Effect of the remnant cutting length on the decomposition process.

**Cutting length of tomato remnants:** As shown in Fig. 1a, the longer tomato remnants were cut, the more residual remnants weight were and total carbon percentage were measured, indicating that the ∼0.5 cm and ∼1.0 cm cutting lengths had a residual remnant weight and total carbon percentage 28.49 % and 32.65 % lower than those of ∼2.0 cm and ∼2.5 cm cuttings.

The decomposition rate and organic carbon breakdown rate showed that both decreased with the increase of the remnant cutting length (Fig. 1b). The decomposition rate and organic carbon breakdown rate of ∼0.5 cm and ∼1.0 cm cuttings were significantly higher than those of ∼2.0 cm and ∼2.5 cm ones, 6.14 % and 7.48 % higher than those of ∼2.5 cm cuttings.

It was obvious that ∼0.5 cm cuttings had the least residual remnant weight and the fastest decomposition rate in comparison with those of other remnant cuttings. In terms of total carbon content and organic carbon breakdown rate, there was no significant difference between ∼0.5 cm and ∼1.0 cm cuttings. However, the organic carbon breakdown rate and decomposition rate of ∼2.0 cm and ∼2.5 cm cuttings were the lowest and slowest.

**Fig. 2:**
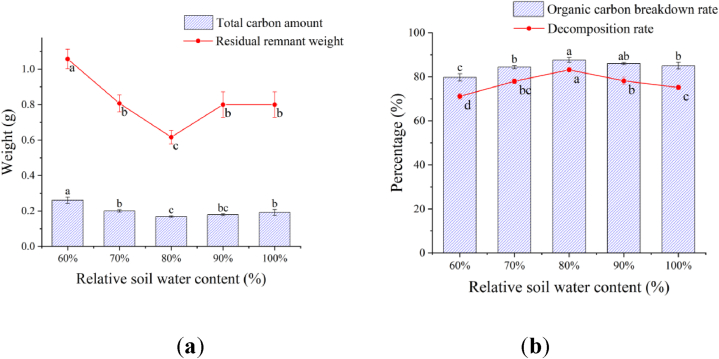
Effect of the soil relative water content on tomato remnant decomposition.

Soil relative water content: With the increase of soil relative water content, the weight of remaining remnants and total carbon amount decreased first and then increased (Fig. 2a) at the highest values with the 60 % of soil relative water content followed by those with the 70 %, 90 %, or 100 % treatments, while the lowest value of those was with the 80 % soil relative water content. The weight of the remaining remnants and total carbon content of 60 % treatment were 1.71 times and 1.55 times higher than those with the 80 % soil relative water content, respectively, showing a significant difference between the treatments.

Both the decomposition rate and organic carbon breakdown rate showed a trend of increment first and then decline later (Fig. 2b). Both measurements with the 80 % of soil relative water content were the highest, followed by those with the 70 %, 90 %, or 100 % treatments. Their lowest measurement occurred with the 60 % treatment. The decomposition rate and organic carbon breakdown rate with the 80 % treatment were 16.94 % and 9.96 % higher than those with the 60 % treatment, respectively.

The highest remnant weight and decomposition rate were significantly found in the treatment with 80 % of soil relative water content. In terms of total carbon content and organic carbon breakdown rate, there was no significant difference between the treatments with 80 % and 90 % of soil relative water content, suggesting extra water (>80 %) in soil is not conducive for tomato remnant decomposition.

**Fig. 3:**
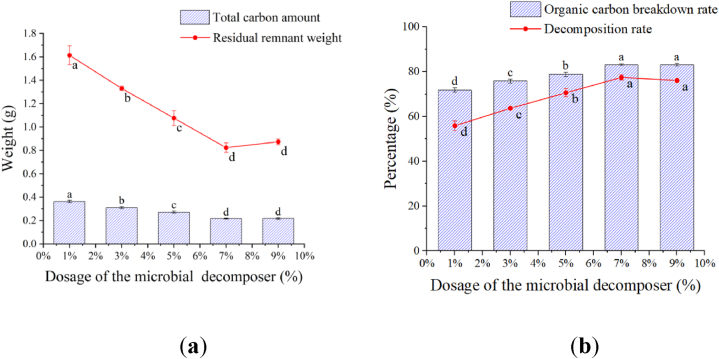
Effect of the microbial decomposer on remnant decomposition.

**Effect of the microbial decomposer on tomato remnant decomposition:** With an increase of the MD amount, the weight and total carbon amount of the remaining remnants decreased (Fig. 3a). The residual remnant weight and total carbon amount were the lowest in the treatments with 7 % and 9 % MD added and the highest in the treatments with 1 % MD added. The residual remnant weight with the 1 % MD treatment was 1.96 times and 1.85 times higher than that of the 7 % and 9 % MD treatments, while the total carbon amount is 1.68 times and 1.67 times higher than that of the 7 % and 9 % MD treatments, respectively. There was no significant difference in total carbon percentage between the 5 %, 7 %, and 9 % MD treatments, but the total carbon percentage with those treatments was significantly higher than that with the 1 % MD treatment.

The decomposition rate and organic carbon breakdown rate increased alone with more MD added (Fig. 3b). There were significant differences among the different MD treatments except those between the 7 % and 9 % MD treatments. Compared with the 1 % MD treatment, the decomposition rate was increased by 38.63 % and 36.19 % with the 7 % and 9 % MD treatments, while the organic carbon breakdown rate increased by 15.89 and 15.78 % with the 7 % and 9 % MD treatments.

**Fig. 4:**
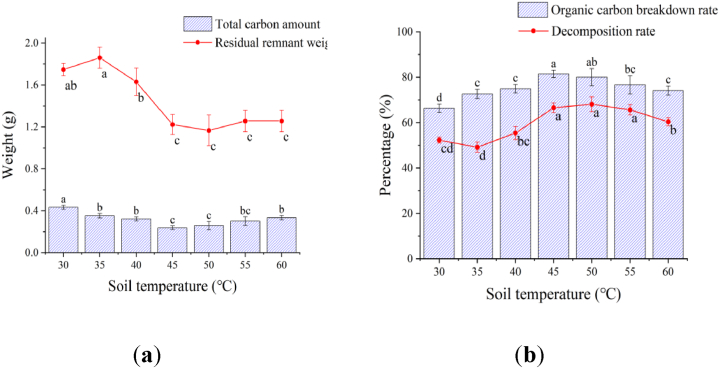
Effect of various soil temperatures on tomato remnant decomposition.

**Effect of soil temperature on tomato remnant decomposition:** With the increase of soil temperature, the residual remnant weight and total carbon decreased first and then increased later (Fig. 4a). The residual remnants weight reached a peak value at 30 °C and 35 °C followed by that at 40 °C, which were significantly higher than those at 45 °C, 50 °C, 55 °C and 60 °C. The total carbon amount was the highest at 30 °C, followed by 35 °C, 40 °C, 55 °C and 60 °C, and the lowest at 45 °C and 50 °C, respectively. The total carbon percentage at 30 °C, 55 °C and 60 °C was significantly higher than that at 35 °C, 40 °C and 45 °C.

There was no significant difference in the decomposition rate among 45 °C, 50 °C and 55 °C treatments, which was significantly higher than other temperature treatments, 27.35 %, 30.31 % and 25.61 % higher than that at 30 °C, respectively (Fig. 4b). The breakdown rate of organic carbon at 45 °C was significantly higher than that of other temperature treatments except that there was no significant difference between 45 °C and 50 °C, and the decomposition rate at 45 °C was 22.78 % higher than that at 30 °C.

In terms of total remnant weight and decomposition rate, there was a significantly difference of the 45 °C, 50 °C, and 55 °C treatments in comparison with other treatments at different temperatures. The results derived from the total carbon amount and organic carbon breakdown rate indicated that temperatures at 45 °C and 50 °C were more conducive for remnant decomposition.

**Equation fitting of single factor decomposition model and optimization of decomposition conditions:** The regression equation of the decomposition rate and organic carbon breakdown rate of was established, indicating that both were linearly related to the remnant cutting length and the amount of the microbial decomposers MD (R2 > 0.877), but not to the soil relative water content and soil temperature (Table 1). According to the regression analysis and limits of the organic carbon decomposition rate, the optimized decomposition of tomato remnants could be achieved when remnants were cut in a length of ∼0.5 cm, soil relative water content kept at 89 %, soil temperature maintained at 50 °C, and 7 % MD added to the remnants.

**Table 1:** Regression equation fitting of the decomposition rate and organic carbon decomposition rate.

Factor	Equation	Decomposition rate (%)			Organic carbon breakdown rate (%)		
		R squared	Sig.	Regression equation	R squared	Sig.	Regression equation
Remnant cutting length	Linear	0.913	0.011	Y = −1.20x + 85.68	0.877	0.019	Y = −1.75x + 89.69
	quadratic	0.997	0.003	/	0.881	0.119	/
Relative soil water content	Linear	0.088	0.627	/	0.426	0.233	/
	quadratic	0.904	0.096	/	0.955	0.045	Y = 2.94 + 1.98x −0.01x2
Soil temperature	Linear	0.517	0.069	/	0.312	0.193	/
	quadratic	0.708	0.085	/	0.931	0.005	Y = −17.55 + 4.05x −0.04x2
Dosage of microbial decomposer	Linear	0.905	0.013	Y = 2.71x + 55.18	0.942	0.006	Y = 1.50x + 71.08
	quadratic	0.98	0.02	/	0.979	0.021	/

Under such optimized decomposition conditions derived from the analysis above, the total weight of remaining remnants and total carbon amount significantly reduced by 99.46 % and 79.00 %, respectively, while the decomposition rate and organic carbon breakdown rate increased by 25.14 % and 11.29 %, respectively (Table 2), in comparison with those under the conditions without optimization (cutting length: ∼2 cm, soil relative water content at 100 %, soil temperature at 40 °C, and 2 % of MD added).

**Table 2:** Comparison of the remnant decomposition efficacy under regular and optimized conditions.

Treatment	Residual remnant weight (g)	Total carbon in residual remnants amount (g)	Decomposition rate (%)	Organic carbon breakdown rate (%)
Regular (CK)	1.23 ± 0.04^a^	21.36 ± 0.01^b^	0.26 ± 0.01^a^	66.48 ± 1.23^b^
Optimized	0.62 ± 0.01^b^	23.8 ± 0.57^a^	0.15 ± 0.06^b^	83.2 ± 0.14^a^

**Principal component analysis of impact factors:** Since the regression model fitting of the organic carbon decomposition rate and four factors were significant, SPSS is used to conduct principal component analysis of the influence of four factors on the organic carbon decomposition rate of tomato remnants.

The KMO and Bartlett tests of four evaluated factors indicated that they were highly correlated (KMO >0.6 and Bartlett p < 0.001), therefore, a factor analysis was performed accordingly (Table 3).

**Table 3:** The Kaiser-Meyer-Olkin and Bartlett tests.

Index		Numerical value
The Kaiser-Meyer-Olkin measure of sample adequacy		0.68
Bartlett's spherical test	Approximate chi2	62.89
	df	6
	Sig.	0.00

Based on the testing results of the factor analysis, the tomato remnant size contributed 83.6 %, soil relative water content 51.3 %, soil temperature 94.2 %，and MD amount 97.0 % of degree of variance in the process of remnant decomposition, respectively (Table 4).

**Table 4:** Variance of common factors.

Factor	Initial value	Extract
Remnant cutting length	1	0.836
Soil relative water content	1	0.513
Soil temperature	1	0.942
Microbial decomposer dosage	1	0.970

However, according to the total variance analysis, only the remnant cutting length had an eigenvalue of 3.261, larger than 1 (Table 5), indicating its major contribution to the tomato remnant decomposition and organic carbon decomposition rate.

**Table 5:** Total variance explained.

Factor	Initial eigenvalue			Extract sum of squares and load		
	Total	Total variance (%)	Accumulation (%)	Total	Total variance (%)	Accumulation (%)
Remnant cutting length	3.261	81.518	81.518	3.261	81.518	81.518
Soil relative water content	0.637	15.924	97.442			
Soil temperature	0.067	1.674	99.116			
Microbial decomposer dosage	0.035	0.884	100.000			

## 4 Discussion

**Remnants Cutting length and decomposition：**Remnants decomposition rate was related to the length of straw cutting length. Shorter straw favorably and significantly decreased soil bulk density, which provided more oxygen to enhance the activity of microorganisms and enzymes. The shorter the straw was cut, the easier it was to decompose [15]. This study has concluded that the decomposition rate and organic carbon decomposition rate decreased with the increase of tomato remnant cutting length and both were negatively correlated. The tomato remnants were decomposed most efficiently when they were cut to the size of ∼0.5 cm long according to the linear regression analysis. Our research findings suggest that the shorter the tomato remnants are cut, the more tightly tomato remnants bind soil to interact with each other during the decomposition process, which is also concluded by Li et al. [11]. The reason is that leguminous remnants have higher nitrogen content than grass plants and is easy to release nutrients in its decomposition.

**Soil relative water content and remnants decomposition：**The effect of soil moisture is of particular importance for plant residue decomposition as it affects the microbial growth and production, activity of microbial extracellular enzymes [16], which are the main drivers of decomposition processes [17]. The amount of water required for their growth and survival of different microorganisms varies greatly [18]. Suitable soil moisture that is optimal for microbial growth and metabolic activities actually accelerates the plant remnant decomposition. Lower decomposition rates are expected both in soils drier and wetter than the optimal soil moisture range [12]. Yet there are also studies that there was no significant negative correlation between straw residue rate and soil moisture content [10]. Our findings confirmed that the tomato remnant decomposition rate was significantly high when the soil relative water content was 80 %. There was no significant difference in terms of the organic carbon decomposition rate between the 80 % and 90 % soil water content treatment. According to the quadratic fitting equation, the optimal soil relative water content for the best organic carbon decomposition is 89 %. Soil moisture content mainly affects the decomposition rate of the plant remnants in the early stage, but may have little effect on the degradation rate in the later stage.

**Microbial decomposers and remnants decomposition：**Many studies have found that the addition of rotten microorganisms to the plant residues returning to the soil will improve the enzyme activity in the plant residues to varying degrees [19], and affect their decomposition rate. Due to the complexity of remnants composition and structure, however, the composite microbial decomposers are more stable and efficient in degradation of plant remnants. The complex strains directly screened from the soil have proven to be stable, effective and synergistic among isolated soil microbes [20]. Jin et al. isolated *Fusarium* sp. F13 and *Microbacterium* sp. B26 isolated from soil, both of which had synergistic effects during co-culture and promoted the decomposition of tomato root litters [21]. In this study, a mixture of complex bacteria and fungi was directly screened and selected from more than 200 soil samples with returned tomato remnants in them. After a series of testing and optimization, the MD mixture of the complex bacteria has demonstrated a highest cellulose degrading power when pH was 7 and the temperature at 50 °C [14]. Although there is a small portion of saprophytic fungi in the MD mixture, most of decaying bacteria are evenly present and function cohesively. The results obtained from this study have also demonstrated that the tomato remnant decomposition rate was the highest when the optimal temperature during the decomposition was around 50 °C. Also, the maximal amount of the MD mixture added is set to be 7 % of total tomato remnant weight, a threshold for the most efficient and balanced decomposition without adding any extra decaying bacteria.

**Fig. 5:**
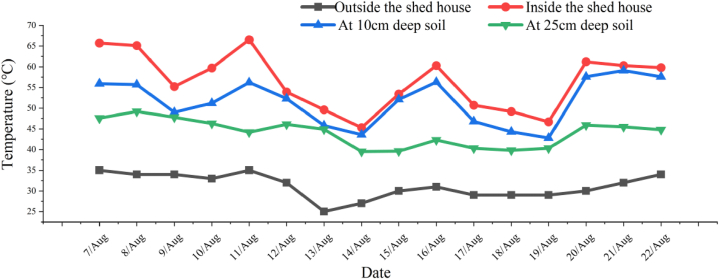
Comparison of the various temperatures during the tomato remnant decomposition.

**Soil temperature and remnants decomposition：**Soil temperature affects the process of plant remnant decomposition and the decomposition rate and organic carbon breakdown accelerate with the increase of soil temperature within a certain range [22]. Stanford et al. has found that the optimal soil temperature for plant remnant decomposition was between 5 and 35 °C, and every 10 °C increase would double the decomposition rate of substances prone to mineralization [23]. By incubation experiment, Chow et al. found that surface peat soils C mineralization rates were 18.3, 40.3, and 49.2 μg per gram at 10 °C, 20 °C and 30 °C respectively on the first day but dropped to 1.5, 5.7, and 9.8 respectively within a week [18]. However, the average soil temperature in a facility is quite different from that in the field due to the fact that it can be controlled within a certain range through the ventilation. The temperature set with this study was adjusted according to the actual measurement of soil temperature in a closed shed house in summer (Fig. 5). As shown in the figure, when the average external temperature is 25°C–35 °C, the temperature inside the shed house can reach 39.6°C–66.5 °C with the average temperature is 42.6°C–57.6 °C at 10 cm deep soil and 39.5°C–49.2 °C at 25 cm deep soil. Therefore, the optimal soil temperature obtained in this experiment is 50 °C, which can be realized, especially the ground temperature 10 cm below the surface.

**Plant remnant decomposition rate and organic carbon conversion:** Mineralization is the biotransformation of organic compounds into simpler inorganic compound [1]. Crop remnant decomposition is a comprehensive process in soil to convert plant debris into simple compounds by mineralization, or to form a new organic matter by humification [24]. Rusnak found that 5–80 % of crop residues were mineralized into useable nutrients for plants, while the remaining 20–25 % of crops were humified, leading to the formation of soil humus [25]. In this study, tomato remnants were all fresh and had less lignification, therefore, the decomposition rate at 15d was from 49.18 % to 85.16 %, and the organic carbon decomposition rate was from 66.36 % to 87.46 % with different treatments, suggesting an efficient and positive conversion of plant material into humus and the organic carbon decomposition rate was higher than the decomposition rate in general. In addition, there is a linear relationship between the organic carbon decomposition rate and four factors evaluated and its difference is significant through the regression model fitting and analysis, indicating the organic carbon decomposition rate may be a better indicator for evaluation of tomato remnant decomposition.

Carbon sequestration and stocks is a double-edged sword to environmental climate change. However, in agricultural production systems, fresh straws contain more dissolved organic carbon and cause a rapid decomposition and mineralization in soil [15]. The easier straws are decomposed and mineralized, the more soil dissolved organic matter are released, which rapidly transform plant remnants into microbe-decomposed products and provide sufficient plant nutrients than those in dried plant remnants [26].

## 5 Conclusions

In conclusion, this study has demonstrated that under the manageable conditions for vegetable production in the facilities, the best and practical way to recycle tomato remnants through decomposition should: 1) cut them into a length of ∼0.5 cm; keep the soil relative content at about 89 %; 3) maintain the soil temperature around 50 °C through adjusting the ventilation; and 4) add 7 % of the MD mixture into tomato remnant cuttings for at least 15 days, which could significantly promote the remnant degradation. Provided certain circumstances the soil temperature or water content cannot be optimized, cutting tomato remnants into small pieces and mixing them with the MD microbial mixture would be the necessary step before returning tomato remnants back to the soil. Compared with a conventional decomposing process, the combination of optimized parameters we proposed here has increased the remnants decay and organic carbon breakdown rate by 25.13%and 11.30 %, respectively. This study provided an efficient decomposition protocol and optimized conditions for returning tomato remnants to facility soil.

## Data availability

All data are original and available upon request. A copy of the dataset was also submitted with the manuscript.

## CRediT authorship contribution statement

**Jingmin Zhang:** Writing – original draft, Investigation, Data curation, Conceptualization. **Wenxia Yang:** Methodology, Investigation. **Di Feng:** Writing – original draft, Validation, Supervision. **Xiaoan Sun:** Writing – review & editing, Writing – original draft.

## Declaration of competing interest

The authors declare that they have no known competing financial interests or personal relationships that could have appeared to influence the work reported in this paper.

## References

[1] Grzyb A., Wolna-Maruwka A., Niewiadomska A. (2020). Environmental factors affecting the mineralization of crop residues. Agronomy.

[2] Chapman S.K., Newman G.S. (2010). Biodiversity at the plant–soil interface: microbial abundance and community structure respond to litter mixing. Oecologia.

[3] Daza Z.T., Gallo A., Rincón L.M., Parrado D.S., Santander M.C., Oviedo A., Chica Martínez, M. M S. (2016). Isolation and characterization of native microorganisms with hydrolytic enzyme activity from sugarcane compost, for bioaugmented processes. Acta Hortic..

[4] Tsegaye B., Balomajumder C., Roy P. (2018). Biodelignification and hydrolysis of rice straw by novel bacteria isolated from wood feeding termite. Biotechnology.

[5] Aulakh M.S., Khera T.S., Doran J.W., Bronson K.F. (2001). Managing crop residue with green manure, urea, and tillage in a rice-wheat rotation. Soil Sci. Soc. Am. J..

[6] Barel J.M., Kuyper T.W., Paul J., de Boer W., Cornelissen J.H.C., De Deyn G.B. (2019). Winter cover crop legacy effects on litter decomposition act through litter quality and microbial community changes. J. Appl. Ecol..

[7] Hassan W., David J., Abbas F. (2014). Effect of type and quality of two contrasting plant residues on CO2 emission potential of Ultisol soil: implications for indirect influence of temperature and moisture. Catena.

[8] Henriksen T., Breland T. (2002). Carbon mineralization, fungal and bacterial growth, and enzyme activities as affected by contact between crop residues and soil. Biol. Fertil. Soils.

[9] Carlesso L., Beadle A., Cook S.M., Evans J., Hartwell G., Ritz K., Sparkes D., Wu L., Murray P.J. (2018). Soil compaction effects on litter decomposition in an arable field: implications for management of crop residues and headlands. Appl. Soil Ecol..

[10] Zhang H., Cao Y., Lyu J. (2021). Decomposition of different crop straws and variation in straw-associated microbial communities in a peach orchard, China. J. Arid Land.

[11] Li X., Li F.Bhupinderpal-Singh, Cui Z., Rengel Z. (2006). Decomposition of maize straw in saline soil. Biol. Fertil. Soils.

[12] Sommers L.E., Gilmour C.M., Wildung R.E., Beck S.M. (1981). The effect of water potential on decomposition processes in soils. Soil Science Society of America, SSSA Special Publicatio.

[13] Kutlu T., Guber A.K., Rivers M.L., Kravchenko A.N. (2018). Moisture absorption by plant residue in soil. Geoderma.

[14] Zhang J.M., Sang M.P., Zhao Z.P., Yang W.X., Wang X.Y. (2022). Optimization of factors affecting cellulase production by soil composite microbial system. Mod. Agric. Sci. Technol..

[15] Zhang Z., Zhang Z., Lu P., Feng G., Qi W. (2020). Soil water-salt dynamics and maize growth as affected by cutting length of topsoil incorporation straw under brackish water irrigation. Agronomy.

[16] Baldrian P. (2014). Distribution of extracellular enzymes in Soils: spatial heterogeneity and determining factors at various scales. Soil Sci. Soc. Am. J..

[17] Moorhead D.L., Lashermes G., Sinsabaugh R.L. (2012). A theoretical model of C- and N-acquiring exoenzyme activities, which balances microbial demands during decomposition. Soil Biol. Biochem..

[18] Chow A.T., Tanji K.K., Gao S., Dahlgren R.A. (2006). Temperature, water content and wet–dry cycle effects on DOC production and carbon mineralization in agricultural peat soils. Soil Biol. Biochem..

[19] Błońska E., Lasota J. (2023). How decaying wood affects the accumulation of polycyclic aromatic hydrocarbons in soil of temperate mountain forest. Environ. Res..

[20] Gong X., Zou H., Qian C., Yu Y., Hao Y., Li L., Wang Q., Jiang Y., Ma J. (2020). Construction of in situ degradation bacteria of corn straw and analysis of its degradation efficiency. Ann. Microbiol..

[21] Jin X., Wang Z.L., Wu F.Z., Li X.G., Zhou X.G. (2022). Litter mixing alters microbial decomposer community to accelerate tomato root litter decomposition. Microbiol. Spectr..

[22] Salah Y.M.S., Scholes M.C. (2011). Effect of temperature and litter quality on decomposition rate of Pinus patula needle litter. Procedia Environmental Sciences.

[23] Stanford G., Frere M.H., Schwaninger D.H. (1973). Temperature coefficient of soil nitrogen mineralization. Soil Sci..

[24] Dijkstra F.A., Zhu B., Cheng W. (2020). Root effects on soil organic carbon: a double‐edged sword. New Phytol..

[25] Rusnak J. (2017). How to improve soil fertility?.

[26] Liang C., Schimel J.P., Jastrow J.D. (2017). The importance of anabolism in microbial control over soil carbon storage. Nat. Microbiol..

